# Characterization of Healthcare-Associated and Community-Associated *Clostridioides difficile* Infections among Adults, Canada, 2015–2019 

**DOI:** 10.3201/eid2806.212262

**Published:** 2022-06

**Authors:** Tim Du, Kelly B. Choi, Anada Silva, George R. Golding, Linda Pelude, Romeo Hizon, Ghada N. Al-Rawahi, James Brooks, Blanda Chow, Jun C. Collet, Jeannette L. Comeau, Ian Davis, Gerald A. Evans, Charles Frenette, Guanghong Han, Jennie Johnstone, Pamela Kibsey, Kevin C. Katz, Joanne M. Langley, Bonita E. Lee, Yves Longtin, Dominik Mertz, Jessica Minion, Michelle Science, Jocelyn A. Srigley, Paula Stagg, Kathryn N. Suh, Nisha Thampi, Alice Wong, Susy S. Hota

**Affiliations:** National Microbiology Laboratory, Winnipeg, Manitoba, Canada (T. Du, G.R. Golding, R. Hizon);; Public Health Agency of Canada, Ottawa, Ontario, Canada (K.B. Choi, A. Silva, L. Pelude, J. Brooks);; British Columbia Children’s Hospital, Vancouver, British Columbia, Canada (G.N. Al-Rawahi);; Alberta Health Services, Calgary, Alberta, Canada (B. Chow);; BC Children’s & Women’s Hospitals, Vancouver (J.C. Collet, J.A. Srigley);; Dalhousie University, Halifax, Nova Scotia, Canada (J.L. Comeau);; Queen Elizabeth II Health Sciences Centre, Halifax (I. Davis);; Kingston Health Sciences Centre, Kingston, Ontario, Canada (G.A. Evans);; McGill University Health Centre, Montréal, Quebec, Canada (C. Frenette);; Provincial Infection Control Network, Vancouver (G. Han);; Sinai Health, Toronto, Ontario, Canada (J. Johnstone);; Royal Jubilee Hospital, Victoria, British Columbia, Canada (P. Kibsey);; North York General Hospital, Toronto (K.C. Katz);; IWK Health Centre, Halifax (J.M. Langley);; Stollery Children’s Hospital, Edmonton, Alberta, Canada (B.E. Lee);; Jewish General Hospital, Montréal (Y. Longtin);; Hamilton Health Sciences, Hamilton, Ontario, Canada (D. Mertz);; Regina General Hospital, Regina, Saskatchewan, Canada (J. Minion);; The Hospital for Sick Children, Toronto (M. Science);; Western Memorial Regional Hospital, Corner Brook, Newfoundland and Labrador, Canada (P. Stagg);; The Ottawa Hospital, Ottawa (K.N. Suh);; Childrens Hospital of Eastern Ontario, Ottawa (N. Thampi);; Royal University Hospital, Saskatoon, Saskatchewan, Canada (A. Wong);; University Health Network, Toronto (S.S. Hota)

**Keywords:** C. difficle, *Clostridioides difficile* infection, bacteria, antimicrobial resistance, healthcare-associated infections, community-associated infections, nosocomial transmission, ribotype, Canada

## Abstract

We investigated epidemiologic and molecular characteristics of healthcare-associated (HA) and community-associated (CA) *Clostridioides difficile* infection (CDI) among adult patients in Canadian Nosocomial Infection Surveillance Program hospitals during 2015–2019. The study encompassed 18,455 CDI cases, 13,735 (74.4%) HA and 4,720 (25.6%) CA. During 2015–2019, HA CDI rates decreased by 23.8%, whereas CA decreased by 18.8%. HA CDI was significantly associated with increased 30-day all-cause mortality as compared with CA CDI (p<0.01). Of 2,506 isolates analyzed, the most common ribotypes (RTs) were RT027, RT106, RT014, and RT020. RT027 was more often associated with CDI-attributable death than was non-RT027, regardless of acquisition type. Overall resistance *C. difficile* rates were similar for all drugs tested except moxifloxacin. Adult HA and CA CDI rates have declined, coinciding with changes in prevalence of RT027 and RT106. Infection prevention and control and continued national surveillance are integral to clarifying CDI epidemiology, investigation, and control.

*Clostridioides difficile* is a major cause of infectious nosocomial diarrhea in high-income countries (*1*). Disease severity ranges from asymptomatic colonization to fulminant colitis, sometimes leading to colectomy and death (*2*). Healthcare costs attributed to *C. difficile* infection (CDI) are estimated to be $4.8 billion in the United States and €3 billion in Europe (*3*). A study in Canada estimated 38,000 annual CDI cases and conservative estimated costs of CDN $280 million resulting from extended hospital stays and rehospitalization (*4*).

The epidemiology of *C. difficile* has evolved markedly in the past decade (*1*). Whereas CDI was once believed to be mostly healthcare-associated (HA), increased evidence points to transmission in community settings (*5*,*6*). An estimated 40% of patients with community-associated (CA) CDI require hospitalization; 20% experience treatment failure, and 28% have recurrent CDI episodes (*7*).

Several international studies have reported changes in molecular and epidemiologic characteristics of CDI in healthcare and community settings (*8*–*13*); we investigated changes in adult CA CDI epidemiology in Canada. The Canadian Nosocomial Infection Surveillance Program (CNISP) collects standardized epidemiologic and laboratory-linked data from sentinel hospitals across Canada, currently representing 30% of all acute care beds. We previously reported a decrease in HA CDI rates during 2009–2015, associated with a reduction in ribotype (RT) 027 (*1*). Here, we describe findings of a multicenter study evaluating incidence, patient characteristics, outcomes, RT prevalence, and antimicrobial resistance rates for HA and CA CDI identified during 2015–2019 in hospitals participating in CNISP. We also assessed associations between predominant RTs and all-cause and CDI-attributable deaths.

## Methods

### Case Definition

We used previously described case definitions for primary CDI (*14*) (Appendix). A case of HA CDI was defined on the basis of laboratory confirmation of CDI and a compatible clinical syndrome developing >72 hours after admission, or <72 hours after admission if the patient had a previous admission to the hospital and was discharged within the previous 4 weeks. CA CDI was defined as clinical manifestation of CDI symptoms <72 hours before admission with no history of hospitalization or healthcare exposure, including outpatient healthcare exposures, within the previous 12 weeks.

Severe outcomes were defined as CDI-attributable admission to an intensive care unit (ICU), colectomy, or death <30 days after admission. All deaths were reviewed by an infectious disease physician or medical microbiologist by using clinical judgement to determine whether deaths were CDI-attributable.

### Data Sources and Collection

CNISP has conducted prospective surveillance for HA CDI in hospitalized patients in Canada since 2007, and CA CDI surveillance was added in 2015. By 2019, CNISP included a network of 76 acute care hospitals across 10 provinces and 1 territory (Appendix Table 1). We analyzed data collected during 2015–2019 from adult and mixed (adult and pediatric) hospitals. The Canadian Network for Public Health Intelligence collected and verified clinical and laboratory surveillance data to ensure accuracy, as previously described (*14*).

### Bacterial Culture and Molecular Characterization

We performed *C. difficile* isolation by using an ethanol shock treatment method, then selected for *C. difficile* on *Clostridium difficile* Moxalactam Norfloxacin agar (Oxoid, https://www.oxoid.com), as previously described (*15*,*16*). We prepared DNA for PCR analysis and ribotyping by using InstaGene Matrix (Bio-Rad, https://www.bio-rad.com), as previously described (*17*). We performed multiplex PCR targeting toxin A (*tcdA*), toxin B (*tcdB*), binary toxin (*cdtB*), negative regulator of toxin production (*tcdC*), and triose phosphate isomerase (*tpi*) housekeeping gene, as previously described (*15*,*18*,*19*), with slight modifications. We substituted an in-house A3B primer (5′-ACCATCAATCTCGAAAAGTCCAC-3′) for the tcd-R reverse primer for detecting *tcdA* (420 bp amplicon) and the detection of *tcdA* deletion variants (147 bp amplicon). 

### PCR Ribotyping

We performed capillary gel electrophoresis–based ribotyping targeting the 16S-23S intergenic spacer region, as previously described (*17*). We assigned RTs by comparing query profiles to those of a reference set of RTs used in a previous multicenter international study (*17*).

### Antimicrobial Susceptibility Testing

We used Etest strips (bioMérieux, https://www.biomerieux.com) to perform susceptibility testing for metronidazole, clindamycin, vancomycin, rifampin, moxifloxacin, and tigecycline, as previously described (*16*,*20*). We interpreted antimicrobial resistance in accordance with Clinical and Laboratory Standards Institute guidelines (*20*).

### Statistical Analysis

We calculated HA CDI incidence rates as number of cases per 10,000 patient-days and CA CDI incidence rates as number of cases per 1,000 patient admissions. We used the Cochran-Armitage test for categorical variables and the Mann-Kendall test for continuous variables to assess statistically significant trends over time for patient characteristics and outcome results. To compare characteristics of patients with HA CDI versus CA CDI, we used the χ^2^ test for categorical variables and the Student *t* test or Wilcoxon rank sum test for continuous variables.

We used multivariable logistic regression to model the association between RTs and outcomes (i.e., 30-day all-cause and 30-day CDI-attributable mortality) and adjusted for a priori–selected confounders of age, sex, severe CDI cases (albumin level <30 g/L, leukocyte count >15 ×10^9^/L, or both), and CDI case types (i.e., HA vs. CA CDI). We used 2-tailed statistical tests and considered p<0.05 statistically significant. We performed all analyses in SAS version 9.4 (SAS Institute Inc., https://www.sas.com).

## Results

Our analysis included a total of 18,455 adult inpatient cases of primary CDI from participating hospitals during 2015–2019. HA CDI accounted for 74.4% (n = 13,735) of cases and CA for 25.6% (n = 4,720). The number of hospitals participating in HA CDI surveillance each year ranged from 58–64, and 46–54 hospitals participated in CA CDI surveillance (Appendix Table 1). We also completed a sensitivity analysis to restrict hospitals that conducted both HA and CA CDI surveillance but observed no stastically significant differences in results (data not shown).

**Figure 1:**
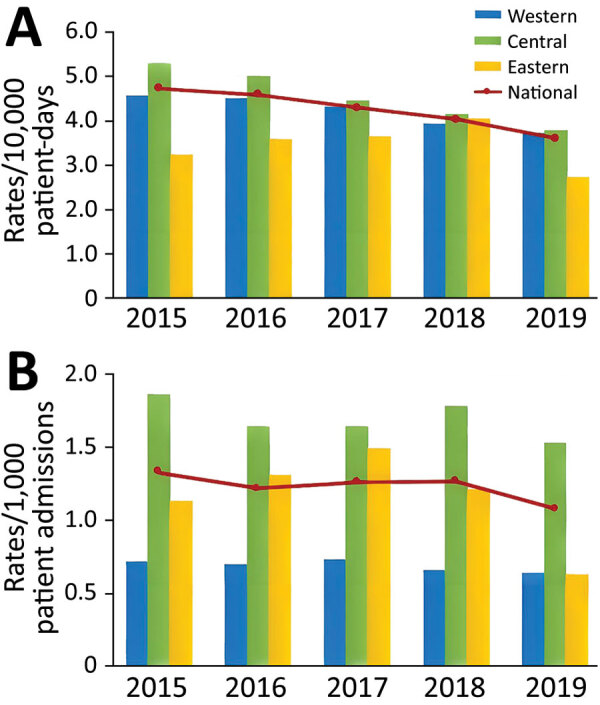
National and regional healthcare-associated (A) and community-associated (B) *Clostridioides difficile* infection rates among adults, Canada, 2015–2019. Western region is British Columbia, Alberta, Saskatchewan, and Manitoba; Central region is Ontario and Quebec; Eastern region is Nova Scotia, New Brunswick, Prince Edward Island, and Newfoundland and Labrador.

During 2015–2019, HA CDI rates decreased by 23.8%, from 4.74 to 3.61 cases/10,000 patient-days (p<0.02), and CA CDI rates decreased by 18.8%, from 1.33 to 1.08 cases/1,000 admissions (p<0.33) (Figure 1). Regionally, HA CDI rates decreased significantly in the central (p<0.02) and western (p<0.02) regions of Canada, but rates fluctuated in the eastern region (p = 0.62), peaking at 4.06 cases/10,000 patient-days in 2019. Despite a decline, CA CDI infection rates remained highest in the central region, at 1.53 cases/1,000 admissions in 2019. Of the 64 hospitals for which data were available for adult CDI surveillance, 58 (91%) reported data for the entire 5-year period of surveillance. After restricting our analyses to these 58 hospitals, interpretation of our results did not change. Incidence rates for HA CDI decreased by 22.8%, CA CDI incidence decreased by 18.0%, and rates were consistent with those reported and generated with data from 64 hospitals.

We aggregated patient characteristics and outcomes by case type (Table 1). Compared with HA CDI patients, CA CDI patients were younger (median age 67.0 vs. 70.0 years; p<0.01), and more CA CDI patients were female (56.0% vs. 49.1% male; p<0.01).

**Table 1:** Clinical and molecular characteristics of healthcare-associated and community-associated *Clostridioides difficile* infection among adults, Canada, 2015–2019*

Characteristics	Healthcare-associated	Community-associated	All cases	p value
Routine surveillance, no. (%)†	13,735 (74.4)	4,720 (25.6)	18,455	
Patient characteristics				
Age, y				
Mean (SD)	68.3 (16.9)	64.4 (18.4)	67.3 (17.4)	**<0.001**
Median (IQR)	70.0 (59.0–81.0)	67.0 (54.0–79.0)	70.0 (58.0–80.0)	**<0.001**
Sex, no. (%)				
F	6,747 (49.1)	2,645 (56.0)	9,392 (50.9)	**<0.001**
M	6,988 (50.9)	2,075 (44.0)	9,063 (49.1)	
Targeted surveillance, no. (%)‡	2,350 (76.2)	734 (23.8)	3,084	
Clinical results and outcomes				
Median (IQR) leukocyte count, × 10^9^ cells/L	10.9 (23.0–33.0)	10.6 (6.9–15.7)	10.8 (7.1–16.0)	NS
Median (IQR) albumin, g/L	26.0 (22.0–31.0)	28.0 (23.0–33.0)	27.0 (22.0–32.0)	**0.0232**
FMT, no. positive/no. tested (%)§	11/3,645 (0.3)	4/1,557 (0.3)	15/5,202 (0.3)	NS
Colectomy, no. positive/no. tested (%)	30/2,255 (1.3)	15/725 (2.1)	45/2,980 (1.5)	NS
Loop ileostomy, no. positive/no. tested (%)	2/798 (0.3)	3/270 (1.1)	5/1,068 (0.5)	NS
ICU admission, no. (%)	n = 2,340	n = 733	n = 3,073	
All cause	156 (6.7)	51 (7.0)	207 (6.8)	NS
Due to complications of CDI	46 (2.0)	11 (1.5)	57 (1.9)	NS
30-d mortality, no. (%)	n = 2,302	n = 731		
Death, all causes	263 (11.4)	53 (7.3)	316/3,033 (10.4)	**0.0001**
Death, attributable to CDI	69 (3.0)	17 (2.3)	86/3,019 (2.9)	NS

*Missing or unknown values were excluded from the analysis. χ^2^ test was used to assess statistical significance for categorical variables; Student *t* test, or the Wilcoxon rank sum test was used for continuous variables. CDI, *Clostridiodes difficile* infection; FMT, fecal microbiota transplantation; ICU, intensive care unit; IQR, interquartile range; NS, not significant. †Patient characeristics data collected year-round. ‡Clinical results and outcome data are collected during a 2-month targeted surveillance period (March–April) each year except FMT where the data were collected year-around. §FMT data collection started in 2018.

### Clinical Manifestations

Of the 18,455 cases, 3,084 had clinical and outcome data available; these data are collected during a 2-month targeted surveillance period (March–April) each year. Overall, 10.4% (316/3,033) of patients with CDI died, and 2.9% (86/3,019) of deaths were CDI-attributable (Table 1). Of 316 deaths among patients with CDI, 27.2% (86/316) were CDI-attributable. Patients with HA CDI had significantly higher 30-day all-cause mortality than patients with CA CDI (11.4% vs. 7.3%; p<0.01). Of 3,073 patients with CDI, 207 (6.8%) required ICU admission, 27.5% (57/207) of whom were admitted because of CDI complications, and 1.9% (57/3,073) all patients with CDI were admitted to the ICU because of CDI complications. We observed no statistically significant differences in ICU admission by acquisition type.

During 2015–2019, ICU admission data were available for 2,340 HA CDI patients (433–507 patients annually). ICU admissions decreased significantly among HA CDI cases, from 9.1% (46/507) in 2015 to 5.9% (26/442) in 2019 (p<0.02). We saw no statistically significant trends for age, sex, or 30-day outcomes for all-cause or CDI-attributable deaths (Appendix Table 2).

### Ribotyping Analysis

Of the 18,455 cases, a total of 3,189 stool samples were received for *C. difficile* isolation at the National Microbiology Laboratory (Winnipeg, Manitoba, Canada), and 2,506 samples met inclusion criteria. Of samples tested, 1,887 (75.3%) were HA CDI and 619 (24.7%) were CA CDI. We performed capillary gel electrophoresis ribotyping and antimicrobial susceptibility testing to further characterize isolates. 

**Figure 2:**
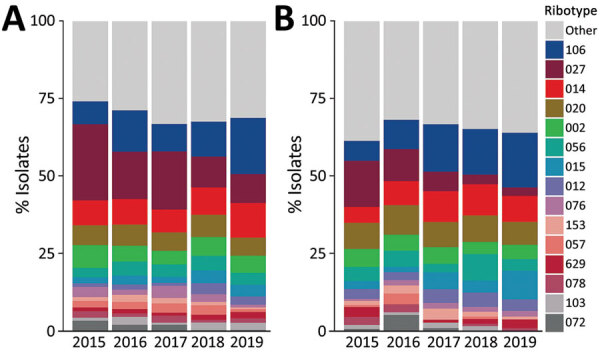
Prevalence of *Clostridioides difficile* ribotypes detected each year from healthcare-associated (A) and community-associated (B) infections among adults, Canada, 2015–2019.

Among 1,887 HA CDI isolates characterized during the study period, we noted 170 unique PCR RTs (Figure 2). The most common RTs among HA CDI were RT027 (16.0%), RT106 (11.5%), RT014 (8.6%), RT020 (6.4%), and RT002 (5.7%). The 15 most prevalent RTs accounted for 69.6% of isolates tested (Appendix Table 3). The prevalence of RT027 in HA CDI cases decreased from 24.6% in 2015 to 9.4% in 2019 (p<0.01), but the incidence of RT106 increased from 7.3% in 2015 to 18.1% in 2019 (p<0.01).

Of 619 CA CDI isolates, we noted 115 unique RTs, of which RT106 (12.3%), RT020 (8.4%), RT014 (8.1%), RT027 (7.9%), and RT056 (5.0%) were the most prevalent. For CA CDI, the 15 most prevalent RTs accounted for 66.1% of isolates tested (Appendix Table 3). As for HA CDI, we noted a decrease in prevalence of RT027, from 14.8% in 2015 to 2.8% in 2019 (p<0.01) and RT106 increased from 6.5% in 2015 to 17.6% in 2019 (p<0.01). Despite a steady decline in prevalence over the study period, RT027 remained the most commonly isolated strain type with an overall combined prevalence of 14.0% (351/2,506 isolates). 

RT078 and RT126 are livestock-associated strains that correlate with increased virulence and disease severity and have been identified in human CDIs. RT078 and RT126 prevalence among HA CDI cases averaged 2.4% (range 2.0%–3.2%), but for CA CDI, RT078 and RT126 prevalence averaged 1.9% (range 0.8%–3.2%) (Appendix Table 4).

### All-Cause and CDI-Attributable Deaths

Among patients with reported 30-day all-cause mortality (n = 316) and 30-day CDI-attributable mortality (n = 86), most were HA CDI: 80.2% (263/316) of all-cause and 83.7% (69/86) of CDI-attributable deaths. In addition, more deaths occurred among female patients, who made up 55.4% (175/316) of all-cause and 57.0% of CDI-attributable (49/86) deaths, and more patients >65 years of age, who comprised 79.8% (252/316) of all-cause and 83.7% of CDI-attributable deaths (72/86).

After multivariable analysis, patient characteristics significantly associated with 30-day all-cause mortality and 30-day CDI-attributable mortality were age >65 years and severe CDI (Table 2). The adjusted odds ratio of 30-day all-cause mortality among patients with HA CDI was 1.83 (95% CI 1.23–2.72) times more than for patients with CA CDI (p<0.01). Similarly, the adjusted odds ratio of 30-day CDI-attributable mortality was 1.25 (95% CI 0.67–2.35) times higher among HA CDI than CA CDI, but this difference was not statistically significant.

**Table 2:** Univariable and multivariable analysis of 30-day all-cause and *Clostridioides difficile*–attributable mortality, Canada, 2015–2019*

Characteristics	Univariable analysis			Multivariable analysis	
	Odds ratio (95% CI)	p value		Adjusted odds ratio (95% CI)	p value
All-cause mortality					
Sex					
M	Referent			Referent	
F	1.15 (0.91–1.45)	0.2484		1.26 (0.93–1.70)	NS
Age group, y					
<65	Referent			Referent	
>65	**2.66 (2.00–3.53)**	**<0.0001**		**3.63 (2.45–5.39)**	**<0.0001**
Severe CDI†	**2.53 (1.90–3.36)**	**<0.0001**		**2.66 (1.90–3.73)**	**<0.0001**
CDI case type					
Community-associated	Referent			Referent	
Healthcare-associated	**1.65 (1.21–2.24)**	**0.0014**		**1.83 (1.23–2.72)**	**0.0028**
RT027 vs. non-RT027	**1.48 (1.04–2.10)**	**0.0289**		1.10 (0.74–1.63)	NS
RT106 vs. non-RT106	1.09 (0.73–1.63)	0.6804		NA	
CDI-attributable mortality					
Sex					
M	Referent			Referent	
F	1.22 (0.79–1.87)	0.3776		1.33 (0.81–1.19)	NS
Age group, y					
<65	Referent			Referent	
>65	**3.28 (1.84–5.85)**	**<0.0001**		**3.44 (1.73–6.82)**	**0.0004**
Severe CDI†	**2.40 (1.45–4.0)**	**0.0006**		**2.25 (1.28–3.94)**	**0.0050**
CDI case type					
Community-associated	Referent			Referent	
Healthcare-associated	1.29 (0.76–2.22)	0.3476		1.25 (0.67–2.35)	NS
RT027 vs. non-RT027	**3.17 (1.89–5.29)**	**<0.0001**		**2.85 (1.64–5.00)**	**0.0002**
RT106 vs. non-RT106	0.95 (0.45–2.00)	0.8830		NA	

*Bold text indicates statistical significance. CDI, *Clostridioides difficile *infection; NA, not applicable; NS, not significant; RT, ribotype. †Severe CDI defined as albumin level <30 g/L, leukocyte count >15 × 10^9^ cells/L, or both.

### Analysis of RT027 and RT106 Outcomes

Among 2,320 case-patients with available data on 30-day all-cause mortality, 316 (13.6%) were reported to have died (Appendix Table 5). Of 235 deaths among patients with associated ribotyping data, 44 (18.7%) deaths were associated with RT027 and 30 (12.8%) deaths with RT106. Among RT027 cases, a significantly higher proportion of 30-day all-cause mortality was associated with HA CDI cases than with CA CDI cases (p = 0.01). We saw no statistically significant difference in 30-day all cause mortality between HA and CA CDI cases associated with RT106. We also saw no statistically significant difference in CDI-attributable deaths when stratified by HA and CA CDI cases for RT027 and RT106.

Of 162 cases with severe outcomes for which ribotype analysis was available in the HA CDI population, 33 (11.7%) were associated with RT027 and 10 (4.8%) were associated with RT106 (p<0.01). We also noted a small number of severe outcomes associated with RT027 (n = 2) and RT106 (n = 3) in CA CDI cases; however, we noted no statistically significant differences between HA and CA CDI.

Multivariate analysis found RT027 was significantly associated with 30-day CDI-attributable mortality (adjusted odds ratio [aOR] 2.85, 95% CI 1.64–5.00) compared with non-RT027 cases (p<0.01). However, the association of RT027 with the outcome of 30-day all-cause mortality did not remain statistically significant compared with non-RT027 cases when controlling for other factors within the multivariate model (aOR 1.10, 95% CI 0.74–1.63). When compared with non-RT106 cases, RT106 was not significantly associated with either 30-day all-cause (p = 0.68) or CDI attributable (p = 0.88) mortality in the univariate model.

### Antimicrobial Susceptibility

**Figure 3:**
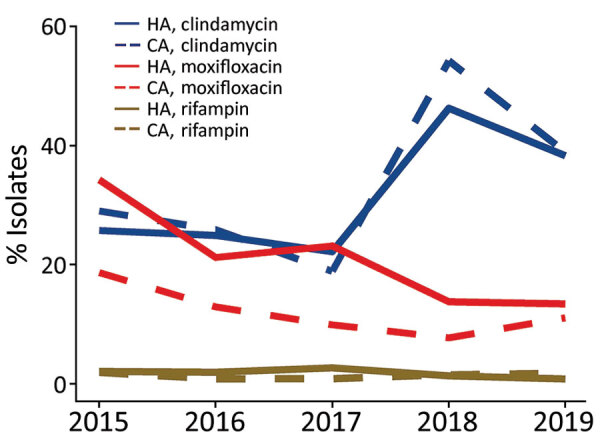
Antimicrobial resistance rates for HA and CA *Clostridioides difficile* infections among adults, Canada, 2015–2019. CA, community-associated; HA, healthcare-associated.

We conducted antimicrobial resistance testing for HA and CA CDI isolates collected during 2015–2019 (Figure 3; Appendix Tables 6, 7). During the study years, HA CDI resistance was 21.7% to moxifloxaxin, 31.0% to clindamycin, and 1.9% to rifampin and CA CDI resistance was 12.4% to moxifloxacin, 33.6% to clindamycin, and 1.5% to rifampin. Of note, HA CDI resistance to moxifloxacin decreased from 34.3% in 2015 to 13.5% in 2019. Similarly, CA CDI resistance to moxifloxacin declined from 18.7% in 2015 to 11.1% in 2019. Resistance to clindamycin was more variable in both study populations, overall resistance was 32.3% (range 19%–54%) (Figure 3).

RT027 accounted for 60.2% (293/487) of identified moxifloxacin-resistant isolates. Of note, 83.5% (293/351) of all RT027 isolates examined were moxifloxacin-resistant, of which 97.3% (285/293) had MICs >32 µg/mL. Among RT027 isolates, resistance was higher in HA CDI (85.4%; 258/302) than CA CDI (71.4%; 35/49) cases. In contrast, RT106, the second most prevelant strain type (11.7%), accounted for 6.0% of all moxifloxacin-resistant isolates. Fluoroquinolone resistance in RT106 isolates was much lower (9.9%; 29/293), and resistance values were similar for both HA (10.6%) and CA settings (7.9%). 

RT027 strains also were more likely to be associated with resistance to >1 antimicrobial drug. Of 172 isolates resistant to both moxifloxacin and clindamycin, 79 (45.9%) were RT027. Of 22 isolates found to be resistant to moxifloxacin, clindamycin, and rifampin, 68.2% (15/22) were RT027; of these, 12 were from HA CDI cases and 3 were from CA CDI cases. No other RT strain exhibited resistance to >1 drug with a prevalence >5%.

We did not observe resistance for metronidazole, vancomycin, or tigecycline for any study year in either HA or CA CDI populations. One adult patient with HA CDI in 2019 had intermediate susceptibility to vancomycin (MIC 6 µg/mL) but sensitivity to all other drugs tested.

## Discussion

Using 5 years of CDI surveillance data from acute care hospitals across Canada, we observed a decline in rates of HA and CA CDI that coincided with a marked change in the prevalence of predominant circulating ribotypes. The epidemiologic and molecular characterization of HA and CA CDI revealed differences in patient characteristics and select clinical outcomes, with associations to predominant ribotypes.

The decline in CDI rates in Canada follows a parallel trend observed globally, despite rates being higher in North America (*10*,*21*). We previously reported HA CDI rates ranging from 2.1 to 6.6 cases/10,000 inpatient days during 2011–2016 but showing a decreasing trend over time (*13*). We noted an increase in CA CDI rates in that study, but in this study, we found that rates of CA CDI have decreased since 2015. Although the precise reasons for decreased CDI incidence in Canada are unclear, enhanced infection control and antimicrobial stewardship measures combined with improved surveillance methods might have contributed to the overall decline (*22*). Furthermore, patients with mild to moderate CA CDI might not be admitted to or tested in a hospital, resulting in underestimation of the true burden of CA CDI.

Although molecular surveillance of CDI in Canada revealed a dynamic and heterogeneous RT population, the predominant circulating types were RT027, RT106, RT020, and RT014. Similar to findings in this study, RT027 has been reported to be decreasing in prevalence in North America, the United Kingdom, and elsewhere; however, RT027 remains a major cause of CDI (*1*,*23*–*26*). In Canada, the dramatic decrease in RT027 prevalence in HA CDI has continued since its initial reporting (*1*). Declining trends observed among HA (−15.2%) and CA (−12.0%) CDI during 2015–2019 in Canada are also consistent with trends in the United States, where HA CDI rates declined from 21% to 15% and CA CDI declined from 17% to 6% during 2012–2017 (*25*).

Although RT027 prevalence in Canada decreased during 2015–2019, RT106 greatly increased during the same period, from 7.3% to 18.1% in healthcare settings and from 6.5% to 17.6% in community settings. Identified in the United Kingdom in 1999 (*27*), RT106 is now found worldwide and is one of the most predominant strains in the United States (*28*). Studies indicate that RT106 has enhanced spore-producing and biofilm formation capabilities that enable greater persistence in the environment and hospital settings, possibly leading to increased infection rates (*28*,*29*). In addition, studies report that patients infected with RT106 are more likely to experience multiple episodes of relapse (*28*,*30*).

*C. difficile* RT078 and RT126, which have demonstrated epidemic potential in other countries (*31*–*33*), appear to be uncommon in patients hospitalized with CDI in Canada. Our surveillance shows a small increase in RT078 and RT126 prevalence since a previous report showed rates of 2.4% in HA and 1.9% CA CDI populations (*14*).

Similar to previous findings, our study showed that CA CDI patients were more likely to be younger and female (*10*,*34*–*36*). In addition, this study found that HA CDI is associated with an increased risk for 30-day all-cause mortality compared with CA CDI; however, this association did not remain significant for CDI-attributable deaths. Hospitalized patients with CDI possibly are exposed to other risks and complications during their hospital stay or have underlying conditions that increase their risk for all-cause death. Our findings agree with previously published studies assessing all-cause and CDI-attributable death (*34*,*37*).

We further analyzed the effects of RT027 and RT106, the 2 most prevalent *C. difficile* strains, on all-cause and CDI-attributable death. We previously showed a significant association between RT027 and attributable mortality (*1*). In this study, we concluded that RT027 is a significant predictor of CDI-attributable death even after adjusting for case type (HA or CA CDI). We noted no association between RT106 and all-cause and CDI-attributable deaths.

We found that *C. difficile* antimicrobial resistance is less common in Canada than in the United States or globally (*38*). Stratified by case type, HA and CA CDI isolates revealed no significant difference in resistance, except for moxifloxacin resistance, which was 21.7% for HA and 12.4% for CA CDI, consistent with previously reported findings (*30*). In addition, diverse RT populations observed in both HA and CA CDI might be predicative of lower resistance rates observed because RT heterogeneity has been shown to be inversely correlated with antimicrobial resistance as measured by cumulative resistance scores (*12*,*39*).

Our study’s first limitations is that hospital participation in HA and CA CDI surveillance varied by year and might have affected trends over time. Furthermore, hospitals self-select whether to participate in both HA and CA CDI surveillance, which might have influenced the comparison of HA and CA CDI patients. To mitigate this limitation, we conducted a sensitivity analysis restricted to hospitals that conducted both HA and CA CDI surveillance. Second, although CDI diagnostic testing methods were collected throughout the study period, data completeness was not consistent from year to year, limiting the inferences we could make regarding the effect of CDI diagnostic testing methods on adult CDI rates over time. Third, for CA CDI surveillance, our study captured data from patients admitted to a CNISP hospital and requiring medical intervention for CDI symptoms or other underlying conditions. The features and outcomes of these patients might not be relevant to patients with CA CDI who do not require hospital care. Finally, although a qualified physician determined the cause of death in patients with CDI, attribution of death is difficult and could be subjective.

In conclusion, rates of HA and CA CDI in Canada declined during 2015–2019, coinciding with a decrease in prevalence of RT027 and increased prevalence of RT106. HA CDI was associated with higher rates of all-cause death than was CA CDI, and RT027 was a major predictor of CDI-attributable death, irrespective of location of acquisition. We noted major decreases in antimicrobial resistance to moxifloxacin in both HA and CA CDI populations, concordant with an overall decrease in prevalence of RT027. Despite declining rates, CDI continues to be a major health burden in Canada. To ensure continued success in combatting this global health threat, robust national surveillance and infection prevention and control programs are integral to clarifying CDI epidemiology, investigation, and control.

Appendix Additional information healthcare-associated and community-associated *Clostridioides difficile* infections among adults, Canada, 2015–2019.
