# Supplementary material for: Characterization of Healthcare-Associated and Community-Associated Clostridioides difficile Infections among Adults, Canada, 2015–2019
Source: Emerg Infect Dis. 2022 Jun;28(6):1128–36. doi: 10.3201/eid2806.212262 (PMC9155897; doi:10.3201/eid2806.212262)
Supplement: Appendix — Additional information healthcare-associated and community-associated Clostridioides difficile infections among adults, Canada, 2015–2019. [file 21-2262-Techapp-s1.pdf]

Article DOI: https://doi.org/10.3201/eid2806.212262

# Characterization of Healthcare-Associated and Community-Associated *Clostridioides difficile* Infections among Adults, Canada, 2015–2019

## Appendix

### Surveillance Case Definitions and Eligibility Criteria

#### *Clostrioides difficile* Infection (CDI)

A "primary" episode of CDI is defined as either the first episode of CDI ever experienced by the patient or a new episode of CDI, which occurs greater than eight weeks after the diagnosis of a previous episode in the same patient.

A patient is identified as having CDI if:

- The patient has diarrhea or fever, abdominal pain and/or ileus AND a laboratory confirmation of a positive toxin assay or positive polymerase chain reaction (PCR) for *C. difficile* (without reasonable evidence of another cause of diarrhea)

OR

- The patient has a diagnosis of pseudomembranes on sigmoidoscopy or colonoscopy (or after colectomy) or histological/pathological diagnosis of CDI

OR

- The patient is diagnosed with toxic megacolon (in adult patients only)

Diarrhea is defined as one of the following:

- More watery/unformed stools in a 36-h period
- or more watery/ unformed stools in a 24-h period and this is new or unusual for the patient (in adult patients only)

Exclusion:

- Any patients younger than one year
- Any pediatric patients (aged one year to younger than 18 y) with alternate cause of diarrhea found (i.e., rotavirus, norovirus, enema or medication, etc.) are excluded even if *C. difficile* diagnostic test result is positive

#### CDI Case Classification

Once a patient has been identified with CDI, the infection will be classified further based on the following criteria and the best clinical judgment of the healthcare and/or infection prevention and control practitioner.

Healthcare-associated (acquired in your facility) CDI case definition

- Related to the current hospitalization
    - The patient's CDI symptoms occur in your healthcare facility three or more days (or $\geq$72 h) after admission
- Related to a previous hospitalization
    - The patient's CDI symptoms occur less than three days after the current admission (or less than 72 h) AND the patient had been previously hospitalized at your healthcare facility and discharged within the previous four weeks
- Related to a previous healthcare exposure at your facility
    - The patient's CDI symptoms occur less than three days after the current admission (or less than 72 h) AND the patient had a previous healthcare exposure at your facility within the previous four weeks

Healthcare-associated (acquired in any other healthcare facility) CDI case definition

- Related to a previous hospitalization at any other healthcare facility
    - The patient's CDI symptoms occur less than three days after the current admission (or less than 72 h) AND the patient is known to have been previously hospitalized at any other healthcare facility and discharged/transferred within the previous four weeks
- Related to a previous healthcare exposure at any other healthcare facility
    - The patient's CDI symptoms occur less than three days after the current admission (or <72 h) AND the patient is known to have a previous healthcare exposure at any other healthcare facility within the previous four weeks

**Community-Associated CDI Case Definition**

The patient's CDI symptoms occur less than three days (or less than 72 h) after admission, with no history of hospitalization or any other healthcare exposure within the previous 12 weeks.

**Appendix Table 1.** Number of Canadian Nosocomial Infection Surveillance Program adult and mixed (adult and pediatric) hospitals in each region included in a study of *Clostridioides difficile* infection among adults, Canada, 2015–2019*

| Region | 2015 | 2016 | 2017 | 2018 | 2019 |
|---|---|---|---|---|---|
| Healthcare-associated | | | | | |
| Western | 22 | 22 | 22 | 22 | 22 |
| Central | 22 | 22 | 23 | 23 | 26 |
| Eastern | 14 | 15 | 15 | 15 | 15 |
| Northern | 0 | 0 | 0 | 0 | 1 |
| All | 58 | 59 | 60 | 60 | 64 |
| Community-associated | | | | | |
| Western | 12 | 12 | 12 | 12 | 12 |
| Central | 20 | 21 | 23 | 23 | 26 |
| Eastern | 14 | 15 | 15 | 15 | 15 |
| Northern | 0 | 0 | 0 | 0 | 1 |
| All | 46 | 48 | 50 | 50 | 54 |

*Western region includes British Columbia, Alberta, Saskatchewan, and Manitoba; Central includes Ontario and Quebec; Eastern includes Nova Scotia, New Brunswick, Prince Edward Island, and Newfoundland and Labrador; and Northern includes Nunavut. The Northern region was included in national rates but not displayed in regional stratification due to zero reported cases of *Clostridioides difficile*.

**Appendix Table 2.** Clinical characteristics and outcome trends of patients with *Clostridioides difficile* infection over time, Canada, 2015–2019*

| Characteristics | 2015 | 2016 | 2017 | 2018 | 2019 | p value |
|---|---|---|---|---|---|---|
| Healthcare-associated *C. difficile* infection | | | | | | |
| Sex, no. (%)† | | | | | | |
| M | 1,487 (50.1) | 1,452 (50.4) | 1,454 (51.7) | 1,326 (49.9) | 1,268 (52.5) | NS |
| F | 1,479 (49.9) | 1,430 (49.6) | 1,360 (48.3) | 1,329 (50.1) | 1,149 (47.5) | NS |
| Median age, y (IQR)† | 71 (59–82) | 70 (58–81) | 71 (59–81) | 70 (59–81) | 71 (59–81) | NS |
| ICU admission, no./total (%)‡ | | | | | | |
| All cause | 46/507 (9.1) | 33/466 (7.1) | 29/492 (5.9) | 22/433 (5.1) | 26/442 (5.9) | **0.0188** |
| Complication from CDI | 13/507 (2.6) | 8/466 (1.7) | 11/492 (2.2) | 5/433 (1.2) | 9/442 (2.0) | NS |
| 30-day outcome death, no./total (%)‡ | | | | | | |
| All cause death | 68/504 (13.5) | 51/442 (11.5) | 49/486 (10.1) | 49/429 (11.4) | 46/441 (10.4) | NS |
| Attributable to CDI | 21/504 (4.2) | 14/442 (3.2) | 16/485 (3.3) | 6/424 (1.4) | 12/438 (2.7) | NS |
| Community-associated *C. difficile* infection | | | | | | |
| Sex, no. (%)† | | | | | | |
| M | 426 (44.0) | 384 (42.7) | 453 (45.9) | 445 (44.6) | 367 (42.3) | NS |
| F | 543 (56.0) | 516 (57.3) | 533 (54.1) | 553 (55.4) | 500 (57.7) | NS |
| Median age, y (IQR)† | 67 (54–79) | 66 (54–79) | 66 (54–78) | 67 (54–78) | 67 (55–79) | NS |
| ICU admission, no./total (%)‡ | | | | | | |
| All cause | 16/164 (9.8) | 11/146 (7.5) | 4/141 (2.8) | 11/145 (7.6) | 9/137 (6.6) | NS |
| Complication from CDI | 3/164 (1.8) | 2/146 (1.4) | 3/141 (2.1) | 1/145 (0.7) | 2/137 (1.5) | NS |
| 30-day outcome death, no./total (%)‡ | | | | | | |
| All cause death | 11/164 (6.7) | 12/146 (8.2) | 7/141 (5.0) | 7/144 (4.9) | 16/136 (11.8) | NS |
| Attributable to CDI | 4/164 (2.4) | 4/146 (2.7) | 1/140 (0.7) | 3/143 (2.1) | 5/133 (3.8) | NS |

*Missing or unknown values were excluded from the analysis. The Cochran–Armitage test was used for categorical variables and the Mann–Kendall test for continuous variables to assess significant trends over time. Bolde text indicates statistical significance. CDI, *Clostridioides difficile* infection; ICU, intensive care unit; NS, not significant.
†Patient demographic characteristics data (age and sex) collected year-round.
‡ICU admission and mortality data are collected during a 2-month targeted surveillance period (March–April) each year.

**Appendix Table 3.** Top 15 most prevalent healthcare-associated and community-associated *Clostridioides difficile* ribotypes among adults, Canada, 2015–2019

| Ribotypes | 2015 | 2016 | 2017 | 2018 | 2019 |
|---|---|---|---|---|---|
| Healthcare-associated, % | | | | | |
| 027 | 24.59 | 15.30 | 18.72 | 9.92 | 9.36 |
| 106 | 7.33 | 13.31 | 8.87 | 11.29 | 18.13 |
| 014 | 8.04 | 8.22 | 7.39 | 8.82 | 11.11 |
| 020 | 6.38 | 6.80 | 5.91 | 7.16 | 5.85 |
| 002 | 7.33 | 5.10 | 4.43 | 6.06 | 5.56 |
| 056 | 3.07 | 4.53 | 3.94 | 4.68 | 3.80 |
| 015 | 1.89 | 2.83 | 1.97 | 4.13 | 3.80 |
| 076 | 3.31 | 1.70 | 3.94 | 2.48 | 0.88 |
| 057 | 2.13 | 2.27 | 2.96 | 3.03 | 0.88 |
| 012 | 1.18 | 1.70 | 0.99 | 3.58 | 2.63 |
| 103 | 0.95 | 2.55 | 0.74 | 2.48 | 2.34 |
| 072 | 3.31 | 1.98 | 1.97 | 0.28 | 0.29 |
| 078 | 2.13 | 1.13 | 2.22 | 0.83 | 1.46 |
| 629 | 1.18 | 1.42 | 0.99 | 1.65 | 2.05 |
| 153 | 1.18 | 2.27 | 1.72 | 1.10 | 0.58 |
| Other | 26.00 | 28.90 | 33.25 | 32.51 | 31.29 |
| Community-associated, % | | | | | |
| 106 | 6.45 | 9.48 | 15.32 | 14.73 | 17.59 |
| 020 | 8.39 | 9.48 | 8.11 | 8.53 | 7.41 |
| 014 | 5.16 | 7.76 | 9.91 | 10.08 | 8.33 |
| 027 | 14.84 | 10.34 | 6.31 | 3.10 | 2.78 |
| 056 | 4.52 | 5.17 | 2.70 | 8.53 | 3.70 |
| 002 | 5.81 | 5.17 | 5.41 | 3.88 | 4.63 |
| 015 | 2.58 | 1.72 | 5.41 | 3.88 | 9.26 |
| 012 | 3.23 | 2.59 | 4.50 | 4.65 | 3.70 |
| 629 | 3.23 | 0.00 | 0.90 | 1.55 | 2.78 |
| 153 | 0.65 | 2.59 | 3.60 | 1.55 | 0.93 |
| 019 | 1.29 | 2.59 | 1.80 | 2.33 | 0.00 |
| 328 | 3.23 | 0.86 | 0.90 | 2.33 | 0.00 |
| 078 | 2.58 | 2.59 | 0.00 | 0.78 | 0.93 |
| 076 | 0.65 | 1.72 | 1.80 | 1.55 | 1.85 |
| 054 | 0.00 | 1.72 | 0.90 | 1.55 | 3.70 |
| Other | 37.42 | 36.21 | 32.43 | 31.01 | 32.41 |

**Appendix Table 4**. Prevalence of livestock-associated *Clostridioides difficile* ribotypes 078/126, Canada, 2015–2019

| Ribotype 078/126 | 2015 | 2016 | 2017 | 2018 | 2019 | 2015–2019 |
|---|---|---|---|---|---|---|
| Healthcare-associated, no. (%) | 10 (2.4) | 7 (2.0) | 13 (3.2) | 9 (2.2) | 7 (2.1) | 2.4 |
| Community-associated, no. (%) | 5 (3.2) | 3 (2.6) | 1 (0.9) | 1 (0.8) | 2 (1.9) | 1.9 |

**Appendix Table 5.** Outcomes of ribotype 027 and ribotype 106 by healthcare-associated and community-associated *Clostridioides difficile* infection, 2015–2019, Canada*

| Ribotypes | All-cause death | | | *C. difficile*–attributable death | | | Severe outcomes† | | |
|---|---|---|---|---|---|---|---|---|---|
| | HA | CA | p value | HA | CA | p value | HA | CA | p value |
| RT027 | 43/277 (15.5) | 1/48 (2.1) | 0.0102 | 22/275 (8.0) | 1/48 (2.1) | 0.2217 | 33/282 (11.7) | 2/48 (4.2) | 0.1348 |
| RT106 | 26/204 (12.8) | 4/73 (5.5) | 0.1227 | 7/204 (3.4) | 1/73 (1.4) | 0.6853 | 10/210 (4.8) | 3/74 (4.1) | 1.0000 |

*Values represent no. deaths/no. cases (%) unless otherwise noted. CA, community-associated; HA, healthcare-associated; RT, ribotype.
†Severe outcomes include CDI attributable ICU admission, colectomy and death combined.

**Appendix Table 6.** $MIC_{50}$ and $MIC_{90}$ of healthcare-associated and community-associated *Clostridioides difficile* infection, Canada, 2015–2019*

| | | 2015 | | | 2016 | | | 2017 | | | 2018 | | | 2019 | | | Study period average | | |
|---|---|---|---|---|---|---|---|---|---|---|---|---|---|---|---|---|---|---|---|
| Ant | Set | $MIC_{50}$ | $MIC_{90}$ | Range | $MIC_{50}$ | $MIC_{90}$ | Range | $MIC_{50}$ | $MIC_{90}$ | Range | $MIC_{50}$ | $MIC_{90}$ | Range | $MIC_{50}$ | $MIC_{90}$ | Range | $MIC_{50}$ | $MIC_{90}$ | Range |
| Mox | HA | 1.5 | >32 | 0.25 to >32 | 1.5 | >32 | 0.38 to >32 | 1.5 | >32 | 0.38 to >32 | 1.5 | >32 | 0.50 to >32 | 1.5 | >32 | 0.50 to >32 | 1.5 | >32 | 0.25 to >32 |
| | CA | 1.5 | >32 | 0.50 to >32 | 1.5 | >32 | 0.38 to >32 | 1 | 3 | 0.38 to >32 | 1.5 | 2 | 0.50 to >32 | 1.5 | 32 | 0.75 to >32 | 1.5 | >32 | 0.38 to >32 |
| Cli | HA | 3 | >256 | 0.125 to >256 | 3 | 256 | 0.047 to 256 | 3 | 96 | 0.094 to >256 | 4 | >256 | 0.25 to >256 | 4 | >256 | 0.25 to >256 | 3 | >256 | 0.047 to >256 |
| | CA | 3 | >256 | 0.094 to >256 | 2 | 256 | 0.094–256 | 3 | 96 | 0.25 to >256 | 6 | 12 | 0.75 to >256 | 3 | 12 | 0.25 to >256 | 3 | 128 | 0.094 to >256 |
| Rif | HA | 0.002 | 0.003 | <0.002 to >32 | 0.002 | 0.003 | <0.002 to >32 | 0.002 | 0.003 | <0.002 to >32 | 0.002 | 0.004 | <0.002 to >32 | 0.002 | 0.003 | <0.002 to >32 | 0.002 | 0.003 | <0.002 to >32 |
| | CA | 0.002 | 0.003 | <0.002 to >32 | 0.002 | 0.003 | <0.002 to >32 | <0.002 | 0.002 | <0.002 to >32 | 0.002 | 0.004 | <0.002 to >32 | 0.002 | 0.004 | <0.002 to >32 | 0.002 | 0.003 | <0.002 to >32 |
| Van | HA | 0.5 | 0.75 | 0.19–3 | 0.5 | 0.75 | 0.25–4 | 0.5 | 0.75 | 0.19–3 | 0.75 | 1 | 0.25–3 | 0.75 | 1 | 0.38–6 | 0.5 | 1 | 0.19–6 |
| | CA | 0.5 | 0.75 | 0.25–1.5 | 0.5 | 0.75 | 0.19–1.5 | 0.5 | 0.75 | 0.25–1.0 | 0.75 | 1 | 0.38–1.5 | 0.75 | 1 | 0.25–2 | 0.5 | 1 | 0.19–2 |
| Met | HA | 0.25 | 1 | 0.032 to 2 | 0.25 | 0.75 | 0.032 to 3 | 0.25 | 1 | 0.023 to 3 | 0.38 | 0.5 | 0.023 to 3 | 0.38 | 0.75 | 0.064 to 4 | 0.38 | 0.75 | 0.023–4 |
| | CA | 0.19 | 0.5 | 0.047–1.5 | 0.25 | 0.75 | 0.047–2.0 | 0.25 | 0.5 | 0.047–2.0 | 0.38 | 0.5 | 0.094–2.0 | 0.38 | 0.75 | 0.047–3.0 | 0.25 | 0.5 | 0.047–3 |
| Tig | HA | 0.064 | 0.125 | <0.016–0.25 | 0.032 | 0.047 | <0.016–0.094 | 0.032 | 0.047 | <0.016–0.38 | 0.032 | 0.047 | 0.016–0.25 | 0.047 | 0.125 | 0.023–0.38 | 0.047 | 0.094 | <0.016–0.38 |
| | CA | 0.064 | 0.125 | <0.016–0.19 | 0.032 | 0.047 | <0.016–0.38 | 0.032 | 0.047 | 0.016–0.094 | 0.047 | 0.047 | 0.016–0.094 | 0.047 | 0.125 | 0.016–0.5 | 0.047 | 0.094 | <0.016–0.5 |

*Values expressed as µg/mL. $MIC_{50}$, MIC required to inhibit the growth of 50% of the isolates; $MIC_{90}$, MIC required to inhibit the growth of 90% of the isolates. Ant, antimicrobial drug tested; CA, community-associated; Cli, clindamycin; HA, healthcare-associated; Met, metronidazole; Mox, moxifloxacin; Rif, rifampin; Set, setting; Tig, tigecycline; Van, vancomycin.

**Appendix Table 7.** Antimicrobial resistance interpretation healthcare-associated and community-associated *Clostridioides difficile* infection, Canada, 2015–2019*

| | 2015 | | | | | | 2016 | | | | | | 2017 | | | | | | 2018 | | | | | | 2019 | | | | | |
|---|---|---|---|---|---|---|---|---|---|---|---|---|---|---|---|---|---|---|---|---|---|---|---|---|---|---|---|---|---|---|
| | HA, n = 423; CA, n = 155 | | | | | | HA, n = 353; CA, n = 116 | | | | | | HA, n = 406; CA, n = 111 | | | | | | HA, n = 363; CA, n = 129 | | | | | | HA, n = 342; CA, n = 108 | | | | | |
| | Sus | | Inter | | Res | | Sus | | Inter | | Res | | Sus | | Inter | | Res | | Sus | | Inter | | Res | | Sus | | Inter | | Res | |
| Ant | No. | % | No. | % | No. | % | No. | % | No. | % | No. | % | No. | % | No. | % | No. | % | No. | % | No. | % | No. | % | No. | % | No. | % | No. | % |
| Mox | | | | | | | | | | | | | | | | | | | | | | | | | | | | | | |
| HA | 273 | 64.5 | 5 | 1.2 | 145 | 34.3 | 271 | 76.8 | 7 | 2.0 | 75 | 21.3 | 312 | 76.9 | 0 | 0 | 94 | 23.2 | 309 | 85.1 | 4 | 1.1 | 50 | 13.8 | 294 | 86.0 | 2 | 0.6 | 46 | 13.5 |
| CA | 126 | 81.3 | 0 | 0.0 | 29 | 18.7 | 97 | 83.6 | 4 | 3.5 | 15 | 12.9 | 99 | 89.2 | 1 | 0.9 | 11 | 9.9 | 117 | 90.7 | 2 | 1.6 | 10 | 7.8 | 93 | 86.1 | 3 | 2.8 | 12 | 11.1 |
| Cli | | | | | | | | | | | | | | | | | | | | | | | | | | | | | | |
| HA | 183 | 43.3 | 131 | 31.0 | 109 | 25.8 | 160 | 45.3 | 105 | 29.8 | 88 | 24.9 | 196 | 48.3 | 120 | 29.6 | 90 | 22.2 | 80 | 22.0 | 115 | 31.7 | 168 | 46.3 | 117 | 34.2 | 94 | 27.5 | 131 | 38.3 |
| CA | 75 | 48.4 | 25 | 22.6 | 45 | 29.0 | 59 | 50.9 | 27 | 23.3 | 30 | 25.9 | 50 | 45.1 | 40 | 36.0 | 21 | 18.9 | 24 | 18.6 | 35 | 27.1 | 70 | 54.3 | 42 | 38.9 | 24 | 22.2 | 42 | 38.9 |
| Rif | | | | | | | | | | | | | | | | | | | | | | | | | | | | | | |
| HA | 412 | 97.4 | 2 | 0.5 | 9 | 2.1 | 346 | 98.0 | 0 | 0 | 7 | 2.0 | 393 | 96.8 | 2 | 0.5 | 11 | 2.7 | 358 | 98.6 | 0 | 0 | 5 | 1.4 | 339 | 99.1 | 0 | 0 | 3 | 0.9 |
| CA | 152 | 98.1 | 0 | 0 | 3 | 1.9 | 115 | 99.1 | 0 | 0 | 1 | 0.9 | 110 | 99.1 | 0 | 0 | 1 | 0.9 | 127 | 98.5 | 0 | 0 | 2 | 1.6 | 106 | 98.2 | 0 | 0 | 2 | 1.9 |
| Tig | | | | | | | | | | | | | | | | | | | | | | | | | | | | | | |
| HA | 423 | 100 | 0 | 0 | 0 | 0 | 353 | 100 | 0 | 0 | 0 | 0 | 406 | 100 | 0 | 0 | 0 | 0 | 363 | 100 | 0 | 0 | 0 | 0 | 342 | 100 | 0 | 0 | 0 | 0 |
| CA | 155 | 100 | 0 | 0 | 0 | 0 | 116 | 100 | 0 | 0 | 0 | 0 | 111 | 100 | 0 | 0 | 0 | 0 | 129 | 100 | 0 | 0 | 0 | 0 | 108 | 100 | 0 | 0 | 0 | 0 |
| Van | | | | | | | | | | | | | | | | | | | | | | | | | | | | | | |
| HA | 423 | 100 | 0 | 0 | 0 | 0 | 353 | 100 | 0 | 0 | 0 | 0 | 406 | 100 | 0 | 0 | 0 | 0 | 363 | 100 | 0 | 0 | 0 | 0 | 341 | 99.7 | 1 | 0.3 | 0 | 0 |
| CA | 155 | 100 | 0 | 0 | 0 | 0 | 116 | 100 | 0 | 0 | 0 | 0 | 111 | 100 | 0 | 0 | 0 | 0 | 129 | 100 | 0 | 0 | 0 | 0 | 108 | 100 | 0 | 0 | 0 | 0 |
| Met | | | | | | | | | | | | | | | | | | | | | | | | | | | | | | |
| HA | 423 | 100 | 0 | 0 | 0 | 0 | 353 | 100 | 0 | 0 | 0 | 0 | 406 | 100 | 0 | 0 | 0 | 0 | 363 | 100 | 0 | 0 | 0 | 0 | 342 | 100 | 0 | 0 | 0 | 0 |
| CA | 155 | 100 | 0 | 0 | 0 | 0 | 116 | 100 | 0 | 0 | 0 | 0 | 111 | 100 | 0 | 0 | 0 | 0 | 129 | 100 | 0 | 0 | 0 | 0 | 108 | 100 | 0 | 0 | 0 | 0 |

*Per Clinical and Laboratory Standards Institute guidelines; Methods for antimicrobial susceptibility testing of anaerobic bacteria, 9th edition. CLSI standard M11. Wayne, PA: The Institute; 2018. Ant, antimicrobial drug; CA, community-associated; HA, healthcare-associated; Inter, intermediate; Res, resistant; Sus, susceptible.

†Values expressed as µg/mL. Antimicrobial susceptibility interpretation: Moxifloxacin (Mox), susceptible ≤2, intermediate = 4, resistant ≥8; clindamycin (Cli), susceptible ≤2, intermediate = 4, resistant ≥8; rifampin (Rif), susceptible ≤1, intermediate = 2, resistant ≥4; vancomycin (Van), susceptible ≤4, intermediate = 8–16, resistant ≥32; metronidazole (Met), susceptible ≤8, intermediate = 16, resistant ≥32; tigecycline (Tig), susceptible ≤4, intermediate = 8, resistant ≥16.
